# A Diagnostic Hint

**Published:** 1889-01

**Authors:** 


A DIAGNOSTIC HINT:—The absence of tears in children four months old or more suggests a form of disease which is usually fatal.

